# Lifetime risk of developing diabetes and years of life lost among those with diabetes in Brazil

**DOI:** 10.7189/jgh.11.04041

**Published:** 2021-07-03

**Authors:** Paula A Bracco, Edward W Gregg, Deborah B Rolka, Maria Inês Schmidt, Sandhi M Barreto, Paulo A Lotufo, Isabela Bensenor, Bruce B Duncan

**Affiliations:** 1 Postgraduate Program in Epidemiology, School of Medicine and Hospital de Clínicas de Porto Alegre, Universidade Federal do Rio Grande do Sul, Porto Alegre, Rio Grande do Sul, Brazil; 2 Department of Diabetes and Cardiovascular Disease Epidemiology, School of Public Health, Imperial College London, UK; 3 Division of Diabetes Translation, Centers for Disease Control and Prevention, Atlanta, Georgia, USA; 4 Department of Preventive and Social Medicine, Medical School, Universidade Federal de Minas Gerais, Belo Horizonte, Brazil; 5 Department of Internal Medicine, School of Medicine, Universidade de São Paulo, São Paulo, Brazil

## Abstract

**Background:**

Given the paucity of studies for low- or middle-income countries, we aim to provide the first ever estimations of lifetime risk of diabetes, years of life spent and lost among those with diabetes for Brazilians. Estimates of Brazil´s diabetes burden consist essentially of reports of diabetes prevalence from national surveys and mortality data. However, these additional metrics are at times more meaningful ways to characterize this burden.

**Methods:**

We joined data on incidence of physician-diagnosed diabetes from the Brazilian risk factor surveillance system, all-cause mortality from national statistics, and diabetes mortality rate ratios from ELSA-Brasil, an ongoing cohort study. To calculate lifetime risk of developing diabetes, we applied an illness-death state model. To calculate years of life lost for those with diabetes and years lived with the disease, we additionally calculated the mortality rates for those with diabetes.

**Results:**

A 35-year-old white adult had a 23.4% (95% CI = 22.5%-25.5%) lifetime risk of developing diabetes by age 80 while a same-aged black/brown adult had a 30.8% risk (95% confidence interval (CI) = 29.6%-33.2%). Men diagnosed with diabetes at age 35 would live 32.9 (95% CI = 32.4-33.2) years with diabetes and lose 5.5 (95% CI = 5.1-6.1) years of life. Similarly-aged women would live 38.8 (95% CI = 38.3-38.9) years with diabetes and lose 2.1 (95% CI = 1.9-2.6) years of life.

**Conclusions:**

Assuming maintenance of current rates, one-quarter of young Brazilians will develop diabetes over their lifetimes, with this number reaching almost one-third among young, black/brown women. Those developing diabetes will suffer a decrease in life expectancy and will generate a considerable cost in terms of medical care.

The prevalence of diabetes has been steadily increasing worldwide, more rapidly in middle- and low-income countries (LMIDs) [1]. For example, in Brazil, the prevalence of self-reported diabetes in capital cities increased from 5.5% in 2006 to 8.9% in 2016 [2], which extrapolates to an additional 450 000 cases nationally every year [3]. Especially for LMICs, spending of governments and societies to treat diabetes, along with the other chronic diseases, will constitute a major challenge to global development in the 21st century [4].

Estimates of Brazil´s diabetes burden, as is the case for most middle- and low-income countries, consist essentially reports of the prevalence of self-reported diagnosed diabetes from national surveys [5] together with mortality data gathered from death certificates [6]. However, additional metrics – lifetime risk of developing diabetes and years of life lost among those with diabetes - are at times more meaningful ways to characterize the diabetes burden. Lifetime risk expresses the probability of an individual without diabetes developing the disease before a certain age and is of special interest because it provides a unique, easily understood perspective of risk, making it suitable for both public health use and for patient education [7,8]. Years of life lost shows how much an individual’s life is likely to be shortened once diabetes has been diagnosed, also providing a more relatable measure considering the individual perspective [9]. Thus, this metric can be helpful to stimulate prevention actions among those at risk of developing the disease.

Lifetime risk of diabetes and years of life lost among those diagnosed have been reported for some high-income countries [10-12] and, among LMICs, to our knowledge only for Mexico [13]. Differences in lifestyle and obesity rates, along with cultural, economic, ethnic and genetic characteristics, make it difficult to generalize previous results to the Brazilian context. Thus, we aim to calculate these metrics for the Brazilian population aged 35-80 by combining longitudinal data from a large Brazilian multicenter cohort study with national mortality statistics and estimates of the prevalence and incidence of physician-diagnosed diabetes.

## METHODS

### Diabetes prevalence and incidence

We estimated age-, ethnicity- and sex-specific prevalence and incidence rates of diagnosed diabetes for the Brazilian population based on publicly available data from the Surveillance System of Risk and Protective Factors for Chronic Diseases by Telephone Survey (Vigitel) [14-16]. This annual telephone survey started in 2006 and focuses on the adult population (18 years or more) living in the 26 state capitals and the Federal District. Vigitel makes use of registers of landline telephone numbers to randomly select its samples. It applies post-stratification weighting based on the 2000 and 2010 demographic censuses to compensate for low and unequal coverage of landline phones, that in 2013 ranged from 75% coverage in the Southeast to 34% in the North [17]. In addition, post-stratification weighting is also applied to compensate for the unequal coverage across age, sex and educational attainment strata [18], and thus to produce representative results. Diagnosed diabetes was defined by the question “Has any doctor ever told you that you have diabetes?”. To estimate incidence, we considered the frequency of cases diagnosed within the last year, by comparing current age and age at diagnosis provided in the question “What age were you diagnosed with diabetes?”. As, on average, respondents will be midway through their current age, the cases where both current age and age at diagnosis were equal, plus half of those cases where this difference was 1, were included. We based our analysis on aggregate data of 157 872 adults between the ages of 35 and 80 obtained in 2017, 2018 and 2019 Vigitel Surveys, and draw conclusions for 2018. Vigitel response rates for these years were 70.0%, 71.1% and 69.2%, respectively. We calculated incidence after excluding cases reporting having a diabetes diagnosis for more than one year and those with missing information on diabetes diagnosis or age of diagnosis, thus our sample was 127 504 adults.

Participants were also requested to characterize their skin color/ethnicity as white, black, brown (“pardo” in Portuguese, implying of mixed ancestry, mostly African and European), yellow (Asian) or indigenous. As Asians and self-reported indigenous constitute very small fractions of respondents, they were excluded from analyses, leaving white and black/brown as ethnicity categories.

We used logistic regression to smooth the age patterns and estimate diabetes age-, ethnicity- and sex-specific prevalence and incidence rates. The model to estimate prevalence included a quadratic term for age (as a continuous variable) and an interaction term between sex and ethnicity. Both quadratic and interaction terms were not significant and therefore not included on the model to estimate incidence. As we found no time trend in diabetes prevalence or incidence over the three years analyzed, we did not incorporate any such trend in our analyses. We used SAS SUDAAN to account for the survey sample design and to produce the weighted average marginal estimates.

### Mortality rates

We estimated the age-, ethnicity- and sex-specific mortality rates for those with and without diabetes by the formula described by Jacobs et al., 2017 [19,20] (see the **Online Supplementary Document**) in two steps. Briefly, this calculation combines Brazilian population projections, ethnicity distributions and all-cause mortality statistics publicly available from the National Institute of Geography and Statistics (IBGE) [21], with the diabetes prevalence estimates from Vigitel. In addition, both the mortality ratio comparing those with self-reported black/brown ethnicity to those who self-reported white and the diabetes mortality rate ratio comparing deaths among those with vs those without diabetes were needed for the formula.

We obtained those mortality rate ratios through Cox regression. We estimated the age- and sex-specific ethnicity mortality rate ratio comparing those that self-reported black/brown ethnicity with those that self-reported white in a model including ethnicity, age and sex. The ethnicity-, age- and sex- specific diabetes mortality rate ratio was obtained on a model including diabetes, age, sex and ethnicity, and adjusted for body mass index (BMI), waist circumference, schooling and income. Both models used data from the Brazilian Longitudinal Study of Adult Health (ELSA-Brasil). ELSA-Brasil is a contemporary cohort study of 15 105 adults initially aged 35-74 [22,23] that ascertained death of participants with and without self-reported diabetes at baseline (2008-2010) through to July 2018 based on annual telephone follow-up. ELSA-Brasil was approved by the institutional review board of the Hospital de Clínicas de Porto Alegre (Approval numbers 06.194 and 1.300.199), and written informed consent from all participants were obtained.

As these mortality rate ratios are calculated from age 35 to 80, our prevalence and incidence estimates, as well as our main results, are presented for that age range. All analyses and graphs were performed in R (R Foundation for Statistical Computing, Vienna, Austria), SAS SUDAAN 9.3 and SAS 9.4 (SAS Institute, Cary NC, USA).

### Lifetime risk and years of life lost

We applied the illness-death model [12,24,25] to calculate both the lifetime risk of developing diabetes and the years of life lost due to diabetes. Our lifetime risk approach estimates the risk of developing diabetes from a defined age up to age 80, conditional to being alive and diabetes-free until that initial age. We applied age-, sex- and ethnicity- specific diabetes incidence rates and mortality rates of the population without diabetes to this model to obtain lifetime risk.

Years of life lost among those with diabetes compares the life expectancy of people with and without diabetes, recognizing that some individuals currently without diabetes will develop it in the future, and thereby decrease their overall probability of survival. The survival of people with diabetes estimates the years a person diagnosed with diabetes is expected to live with the condition and is calculated using diabetes mortality rates. The survival of people currently without diabetes utilizes not only the probability of their not dying before reaching given ages but also the probabilities of acquiring diabetes at these ages and, if acquired, of surviving with diabetes afterwards. Therefore, to calculate the years of life lost we combined diabetes incidence rate estimates with the mortality rates of those with and without diabetes [25].

Both lifetime risk and years of life lost are cumulative estimates calculated by inserting incidence and mortality rates into integrals of functions derived from standard probability theory (Appendix S1 in the **Online Supplementary Document**). For each ethnicity and sex, starting at age 35, we obtained lifetime risk and years of life lost across age intervals as the cumulative sum of calculations for all individual years of the interval (eg, estimates to 40 years are the cumulative result of calculations from age 35 to 40).

Our estimates of lifetime risk and years of life lost are calculated from the period of the three Vigitel surveys rather than being based on a cohort followed through time. Given this perspective, outcomes reported in this work are best considered period rather than cohort estimates, for example, years of life lost are period expected years of life lost [26].

We estimated uncertainty through simulation and bootstrapping (see the **Online Supplementary Document**). All calculations were programmed with R.

## RESULTS

Except for the diabetes mortality ratio, none of our estimates was adjusted for anthropometric or socioeconomic factors because we were interested in observe the crude size of the population diabetes burden. We provide (Table S1 in the **Online Supplementary Document**) the characteristics of self-reported white, or black/brown women and men in Vigitel. Among both men and women, those black/brown presented a significantly worse self-assessment of their own health, a lower frequency of private health insurance and a greater one of receiving government cash transfer, lower education, and less intake of fruits or vegetables (*P* < 0.001). In addition, self-reported whites presented a higher frequency of glucose testing within the last year, although not a significantly higher one among women. Among both ethnicities, women reported more frequent glucose testing then men.

**Figure 1:**
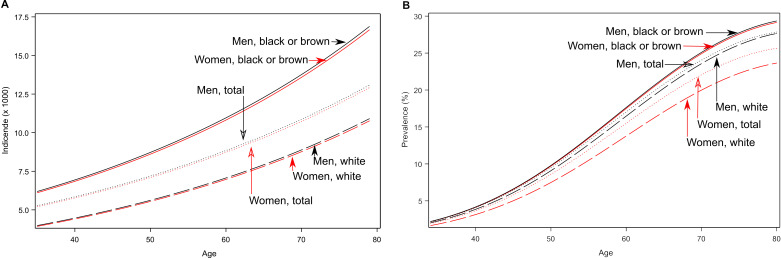
Incidence and prevalence of diagnosed diabetes for Brazilian men and women, self-reported either as white or as black/brown, aged 35-80 years. Panel A. Incidence (per 1000 people). Panel B. Prevalence (%).

The overall self-reported Vigitel incidence (/1000) of diagnosed diabetes was 6.92 (95% CI = 3.95-12.12) and 6.86 (95% CI = 3.92-12.01) for white and 10.63 (95% CI = 5.76-19.56) and 10.55 (95% CI = 6.27-17.78) for black/brown men and women, respectively. This overall sex/ethnicity pattern was relatively constant across the 35 to 80 years age range, with similar incidence of diagnosed diabetes between women and men and a consistently, although not significantly, higher incidence among those black/brown than white (**Figure 1**, Panel B).

The overall self-reported prevalence of known diabetes was 10.3% (95% CI = 9.3%-11.3%) and 9.9% (95% CI = 9.1%-10. 7%) for white men and women, and 11.3% (95% CI = 10.3%-12.4%) and 10.4% (95% CI = 9.6%-11.3%) for black/brown men and women, respectively. As shown in **Figure 1**, Panel B, the difference in prevalence between ethnicities increases at older ages, especially for women, among whom this difference is significant (*P* < 0.001).

**Figure 2:**
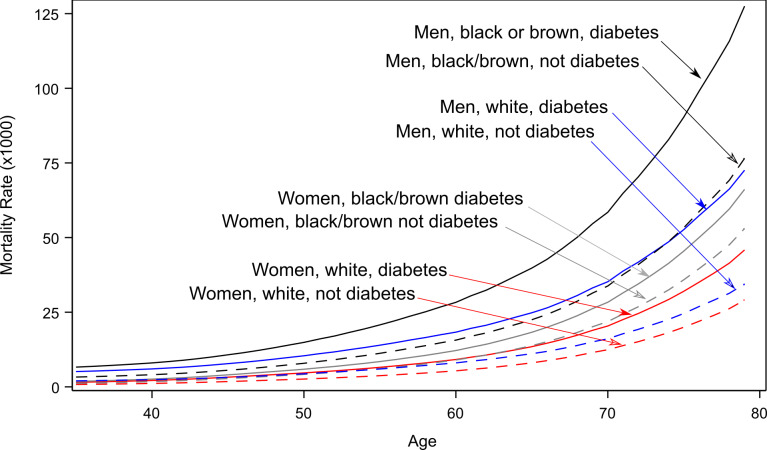
Mortality rate (per 1000 people) of individuals aged 35-80 years with (solid lines) and without (dashed lines) known diabetes. For men and women, self-reported as either white or as black/brown.

White women presented the lowest mortality rate. For of black/brown women, those with and without diagnosed diabetes presented similar rates. Among men, this ethnic difference was not observed. Men with diagnosed diabetes presented higher mortality rates, with the highest rate being observed among self-reported black/brown men with diabetes (**Figure 2**). In addition, a greater difference in mortality rates between those with and without diabetes was present in men than in women. The rate ratio for diabetes in white men (MRR = 2.36; 95% CI = 1.66-3.09) was somewhat higher than that of black/brown men (MRR = 1.80; 95% CI = 1.37-2.38). White women also presented a higher diabetes mortality rate ratio (MRR = 1.69; 95% CI = 1.12-2.57) than black/brown women (MRR = 1.35; 95% CI = 0.94-1.93), for whom the difference in mortality was not statistically significant.

**Table 1** and **Table 2** show lifetime risk of developing known diabetes, years of life lost among those with the condition and years lived with diabetes. For white women without diabetes at age 35 the risk of developing diabetes before age 50 was 7.1% (95% CI = 6.4%-8.6%), and for black/brown women 10.8% (95% CI = 9.9%-12.7%). Considering risk up to the age of 80, 23.8% (95% CI = 22.9%-26.5%) of white women and 32.2% (95% CI = 31.2%-35.2%) of black/brown women would be expected to develop diabetes. The estimates for men were similar although slightly lower: by age 80, 23.0% (95% CI = 21.8%-25.7%) of white men and 29.3% (95% CI = 27.9%-33.0%) of black/brown men would be expected to develop diabetes.

**Table 1:** Mortality with and without diabetes, incidence of diabetes, lifetime risk (LTR) of developing diabetes and years of life lost (YLLs) among those with diabetes and years lived with diabetes (YLDM). Brazil, 2018, by gender and by self-reported ethnicity*

	Women						Men					
**Age**	**Mortality of those without diabetes (×1000)**	**Mortality of those with diabetes (×1000)**	**Incidence (×1000)**	**LTR (%)†**	**YLLs**‡** (years)**	**YLDMs**§	**Mortality of those without diabetes (×1000)**	**Mortality of those with diabetes (×1000)**	**Incidence (/1000)**	**LTR (%)†**	**YLLs**‡** (years)**	**YLDMs**§	
235	1.02 (0.99-1.04)	1.61 (0.86-3.03)	5.20 (2.94-9.18)		2.13 (1.86-2.56)	38.71 (38.30-38.92)	2.66 (2.54-2.73)	5.69 (3.12-10.20)	5.27 (2.66-10.43)		5.53 (5.14-6.11)	32.89 (32.38-33.23)	
40	1.47 (1.41-1.51)	2.28 (1.27-4.06)	5.77 (3.53-9.41)	3.21 (2.67-4.13)	2.12 (1.85-2.55)	33.73 (33.34-33.95)	3.24 (3.07-3.35)	6.83 (4.17-10.83)	5.85 (3.18-10.72)	3.24 (2.46-4.39)	5.49 (5.12-6.06)	27.98 (27.49-28.31)	
50	3.38 (3.17-3.53)	5.09 (3.57-7.28)	7.10 (4.87-10.34)	9.15 (8.38-10.44)	2.06 (1.81-2.46)	23.99 (23.63-24.19)	6.02 (5.57-6.34)	12.32 (9.21-16.49)	7.19 (4.41-11.71)	9.11 (7.99-10.91)	5.17 (4.84-5.64)	18.70 (18.31-18.97)	
60	7.08 (6.46-7.50)	10.35 (8.07-13.56)	8.73 (6.10-12.57)	15.68 (14.91-17.31)	1.84 (1.62-2.19)	14.74 (14.44-14.91)	11.46 (10.66-12.18)	22.78 (19.41-26.73)	8.84 (5.66-13.79)	15.4 (14.08-17.44)	4.32 (4.06-4.66)	10.52 (10.25-10.71)	
70	16.82 (14.92-18.58)	23.89 (17.63-31.08)	10.73 (6.75-17.01)	22.44 (21.55-24.33)	1.28 (1.12-1.51)	6.45 (6.25-6.57)	23.76 (20.94-26.35)	45.85 (37.41-54.36)	10.87 (6.61-17.83)	21.61 (20.33-23.96)	2.67 (2.51-2.88)	4.05 (3.90-4.16)	
80	38.87 (31.85-44.18)	53.76 (37.20-73.49)	12.92 (7.10-23.48)	28.02 (27.12-30.24)			49.84 (40.45-59.35)	93.66 (69.19-119.66)	13.1 (7.15-23.87)	26.38 (24.98-28.91)			
	**White**						**Black/Brown**						
**Age**	**Mortality of those without diabetes**	**Mortality of those with diabetes**	**Incidence**	**LTR (%)†**	**YLLs**‡** (years)**	**YLDMs**§	**Mortality of those without diabetes**	**Mortality of those with diabetes**	**Incidence**	**LTR (%)†**	**YLLs**‡** (years)**	**YLDMs**§	
	**(×1000)**	**(×1000)**	**(/1000)**	**(%)**	**(years)**	**(years)**	**(×1000)**	**(×1000)**	**(/1000)**	**(%)**	**(years)**	**(years)**	
35	1.41 (1.36-1.46)	3.33 (1.63-6.34)	3.95 (2.08-7.49)		4.13 (3.91-4.72)	36.85 (36.15-36.92)	2.24 (2.15-2.83)	4.12 (2.35-7.46)	6.15 (3.34-11.30)		4.15 (3.77-4.69)	34.06 (33.76-34.48)	
40	1.76 (1.67-1.86)	4.08 (2.48-7.40)	4.43 (2.54-7.74)	2.46 (1.99-3.29)	4.10 (3.89-4.69)	31.90 (31.23-31.97)	2.91 (2.73-2.97)	5.22 (3.16-8.12)	6.89 (4.05-11.71)	3.79 (2.98-4.96)	4.13 (3.76-4.66)	29.13 (28.84-29.54)	
50	3.40 (3.26-3.69)	7.51 (5.33-10.65)	5.58 (3.61-8.60)	7.14 (6.56-8.40)	3.87 (3.68-4.39)	22.34 (21.75-22.56)	6.06 (5.63-6.23)	10.37 (7.77-13.49)	8.66 (5.71-13.12)	10.74 (9.60-12.40)	3.94 (3.60-4.40)	19.69 (19.48-20.08)	
60	6.41 (5.90-7.15)	13.50 (11.15-16.93)	7.01 (4.70-10.44)	12.48 (11.75-13.99)	3.28 (3.14-3.70)	13.49 (13.01-13.65)	12.38 (11.25-12.88)	20.25 (16.20-23.74)	10.88 (7.30-16.18)	18.20 (17.03-20.14)	3.38 (3.12-3.72)	11.21 (11.09-11.54)	
70	13.33 (11.80-15.41)	26.82 (22.54-33.40)	8.81 (5.49-14.12)	18.27 (17.45-19.95)	2.10 (2.00-2.34)	5.80 (5.50-5.90)	27.92 (24.24-29.92)	43.59 (33.67-52.21)	13.66 (8.43-22.15)	25.47 (24.16-27.70)	2.18 (2.01-2.39)	4.35 (4.29-4.56)	
80	29.10 (24.28-35.87)	56.15 (43.69-78.06)	10.82 (5.93-19.68)	23.27 (22.46-25.52)			65.04 (49.97-72.94)	97.36 (68.55-121.64)	16.76 (9.00-30.98)	30.74 (29.55-33.15)			

*Numbers in parentheses are 95% CI.

†LTR – lifetime risk: risk of a healthy 35 year-old of developing diabetes before the stated age.

‡YLLs – numbers of years lost in comparison to an individual without diabetes, from the stated age up to 80 years.

§YLDMs – years lived with diabetes, ie, expected life years lived with diabetes, when diagnosed at the stated age and up to age of 80.

**Table 2:** Mortality with and without diabetes, incidence of diabetes, lifetime risk (LTR) of developing diabetes, years of life lost (YLLs) among those with diabetes, and years lived with diabetes (YLDM). Brazil, 2018 by gender and self-reported ethnicity*

	White women						Black/brown women					
**Age**	**Mortality of those without diabetes**	**Mortality of those with diabetes**	**Incidence**	**LTR†**	**YLLs**‡	**YLDMs**§	**Mortality of those without diabetes**	**Mortality of those with diabetes**	**Incidence**	**LTR†**	**YLLs**‡	**YLDMs**§
	**(×1000)**	**(×1000)**	**(/1000)**	**(%)**	**(years)**	**(years)**	**(×1000)**	**(×1000)**	**(/1000)**	**(%)**	**(years)**	**(years)**
35	0.79 (0.77-0.81)	1.52 (0.75-3.05)	3.93 (1.98-7.78)		2.33 (2.08-2.77)	39.42 (38.84-39.72)	1.22 (1.17-1.24)	1.84 (0.96-3.50)	6.12 (3.28-11.33)		2.19 (1.88-2.67)	37.66 (37.31-38.03)
40	1.13 (1.08-1.16)	2.11 (1.14-3.78)	4.41 (2.39-8.12)	2.46 (1.94-3.36)	2.32 (2.07-2.76)	34.45 (33.87-34.76)	1.76 (1.67-1.81)	2.62 (1.51-4.58)	6.85 (3.96-11.83)	3.78 (3.09-5.01)	2.18 (1.88-2.66)	32.69 (32.23-33.05)
50	2.55 (2.34-2.68)	4.56 (2.92-7.15)	5.55 (3.32-9.24)	7.14 (6.38-8.55)	2.23 (2.00-2.64)	24.68 (24.13-24.87)	4.24 (3.91-4.45)	5.99 (3.87-8.20)	8.61 (5.48-13.50)	10.79 (9.91-12.68)	2.13 (1.84-2.59)	22.98 (22.57-23.34)
60	5.20 (4.67-5.62)	8.89 (6.67-12.51)	6.98 (4.26-11.39)	12.54 (11.69-14.44)	1.96 (1.75-2.30)	15.37 (14.91-15.63)	10.06 (9.06-10.94)	13.56 (9.07-15.72)	10.82 (6.91-16.90)	19.25 (17.60-21.55)	1.89 (1.67-2.29)	13.00 (12.62-14.16)
70	11.96 (10.28-13.33)	19.59 (15.05-27.58)	8.77 (4.99-15.35)	18.49 (17.53-20.72)	1.32 (1.17-1.55)	6.89 (6.58-7.02)	22.47 (18.96-24.32)	29.12 (20.82-36.66)	13.58 (7.92-23.19)	26.21 (25.20-28.82)	1.35 (1.16-1.60)	5.87 (5.63-6.08)
80	27.83 (22.69-32.54)	43.86 (30.01-65.24)	10.77 (5.45-21.15)	23.82 (22.89-26.50)			54.99 (41.71-61.34)	68.57 (44.14-93.94)	16.67 (8.52-32.34)	32.16 (31.19-35.17)		
	**White men**						**Black/brown men**					
**Age**	**Mortality of those without diabetes**	**Mortality of those with diabetes**	**Incidence**	**LTR†**	**YLLs**‡	**YLDMs**§	**Mortality of those without diabetes**	**Mortality of those with diabetes**	**Incidence**	**LTR†**	**YLLs**‡	**YLDMs**§
	**(×1000)**	**(×1000)**	**(/1000)**	**(%)**	**(years)**	**(years)**	**(×1000)**	**(×1000)**	**(/1000)**	**(%)**	**(years)**	**(years)**
35	2.00 (1.90-2.07)	5.08 (2.68-9.59)	3.98 (1.91-8.29)		5.43 (5.14-6.12)	34.71 (33.90-34.93)	3.30 (3.15-3.37)	6.65 (3.35-12.02)	6.19 (2.94-12.99)		5.70 (5.17-6.40)	30.80 (30.37-31.33)
40	2.38 (2.26-2.52)	5.92 (3.49-9.94)	4.47 (2.31-8.63)	2.48 (1.86-3.49)	5.39 (5.10-6.06)	29.79 (20.01-29.99)	4.10 (3.81-4.25)	8.10 (5.00-13.78)	6.94 (3.53-13.61)	3.80 (2.88-5.24)	5.65 (5.13-6.34)	25.90 (25.48-26.42)
50	4.26 (2.93-4.66)	10.16 (7.34-14.67)	5.62 (3.26-9.66)	7.15 (6.26-8.74)	5.04 (4.79-5.63)	20.41 (19.74-20.69)	8.02 (7.42-8.34)	15.13 (10.71-19.99)	8.72 (4.89-15.51)	10.70 (9.31-13.12)	5.32 (4.86-5.88)	16.76 (16.43-17.21)
60	7.76 (7.18-8.74)	17.73 (14.76-22.68)	7.07 (4.29-11.63)	12.45 (11.41-14.37)	4.20 (4.01-4.65)	11.97 (11.47-12.00)	15.99 (14.27-16.89)	28.91 (23.03-33.85)	10.96 (6.31-18.98)	17.93 (16.59-21.00)	4.43 (4.08-4.85)	8.93 (8.74-9.29)
70	15.43 (13.72-18.31)	32.77 (27.85-42.57)	8.88 (5.14-15.30)	18.12 (17.07-20.39)	2.43 (2.49-2.87)	4.94 (4.64-5.05)	34.70 (29.71-37.68)	60.07 (47.06-69.92)	13.76 (7.50-25.14)	24.71 (23.30-28.12)	2.70 (2.50-2.95)	3.12 (3.03-3.34)
80	32.93 (27.27-41.3)	69.32 (53.00-93.24)	10.91 (5.70-20.78)	23.05 (21.82-25.72)			79.43 (59.83-91.22)	132.34 (94.08-168.85)	16.89 (8.29-34.07)	29.29 (27.93-32.99)		

*Numbers in parentheses are 95% CI.

†LTR – lifetime risk: risk of a healthy 35 year-old of developing diabetes before the stated age.

‡YLLs – numbers of years lost in comparison to an individual without diabetes, from the stated age up to 80 years.

§YLDMs – years lived with diabetes, ie, expected life years lived with diabetes, when diagnosed at the stated age and up to age of 80.

Considering life expectancy to 80 years, a self-reported white man diagnosed with diabetes at age 35 would lose 5.4 (95% CI = 5.1-6.1) years of life and a similarly-aged self-reported black/brown man 5.7 (95% CI = 5.2-6.4) years of life. The burden was lower for women with diabetes, with 2.3 (95% CI = 2.1-2.8) and 2.2 (95% CI = 1.9-2.7) years of life lost among those with diabetes for a 35-year-old self-reported as white and as black/brown, respectively. On the other hand, women are expected to live longer with the condition. A white woman diagnosed with diabetes at age 35 would be expected to live 39.4 years (95% CI = 38.8-39.5) and a black/brown, 37.7 years (95% CI = 37.3-38.0) with diabetes, as opposed to 34.7 years (95% CI = 33.9-34.8) and 30.8 years (95% CI = 30.4-31.3) in white and black/brown men, respectively.

## DISCUSSION

Our results demonstrate, in easily understood terms, the enormous burden diabetes will cause Brazilians in the foreseeable future if the current scenario is maintained. We estimate that young self-reported black/brown Brazilian adults, in 2018, living to age 80 will have more than a 1 chance in 4 of developing diabetes, and young white Brazilian adults more than a 1 chance in 5. As for the mortality burden, the difference was greater in men, and less affected by ethnicity. We estimated women diagnosed with diabetes at age 35 will lose 2.1 years of life, while men diagnosed at the same age will lose 5.5 years of life. In addition to this loss of life, diabetes will produce an enormous cost in terms of medical care, as, for example, women who develop diabetes at 35 will live, on average, almost four decades with the disease, and men approximately three decades. While our data did not allow for discrimination between type 1 and type 2 diabetes, since we used cases starting from age 35 they are likely nearly all cases of type 2 disease, an eminently preventable disease.

As seen in **Table 3**, the lifetime risks for diabetes in our study are somewhat less for men but more for women than those estimated for high-income countries [10-12]. Regarding LMICs, the only estimates of lifetime risk we found were for Mexico [13], which were considerably higher than all other estimates, in consonance with the greater overall prevalence of obesity and diabetes, and greater diabetes mortality in Mexico [27].

**Table 3:** Comparison of findings on lifetime risk of diabetes and years of life lost among those with diabetes with similar findings of other countries [10-13]

Lifetime risk			Years of life lost		
	**Women**	**Men**		**Women**	**Men**
Brazil – Healthy at 35 (2017-2019)	41.3%	28.0%	Brazil – Diagnosed at 40	3.1	6.1
USA – Healthy at 40 (2000-2011)	36.0%	37.9%	USA – Diagnosed at 40	6.8	5.8
Denmark – Through life (1995-2006)	30.0%	32.0%			
Australia – Healthy at 25 (2000-2005)	36.7%	39.9%	Australia –Diagnosed at 45	4.9	5.5
Mexico – Through life (2010)	57.7%	48.8%			

That black/brown Brazilians had 7.5 percentage point greater lifetime risk, related principally to their greater self-reported incidence of diabetes, supports the importance of considering health disparities as one of the root causes of diabetes when planning prevention programs [28]. Higher lifetime risk among those with non-white ethnicity was also seen for the US, with the risk among non-Hispanic blacks reaching 42% for a 40-year old man and a 51.8% for a 40-year old woman. Black/brown Brazilians with diabetes also had greater mortality rates. However, mortality rates of black/brown Brazilians without diabetes were also higher, almost in the same proportion. This results in a similar burden of years of life lost among those with diabetes for both ethnicities, but leads a 35-year old self-reported black/brown individual diagnosed with diabetes to be expected to live close to 2.5 years less than a self-reported white adult diagnosed at the same age.

The greatest disparity we observed in the diabetes mortality burden was between men and women. Considering a life expectancy of 80 years, the loss in future life expectancy for a 40-year man with diabetes was more than double that of a similarly-aged woman: 5.5 vs 2.1 years. Differences of this size were not seen for the United States [10] and Australia [11]. Compared to our findings, the larger estimates of years of life lost for Australian and United States women could be due to the fact that women, more than men, frequently live beyond age 80, the cut off age for our calculations. They could also result from the fact that estimates in these countries were for earlier time periods – 2000-2011 for the United States and 2000-2005 for Australia, compared to 2017-2019 for our study, as all-cause diabetes mortality has decreased notably over recent years in both countries [29,30].

Our lower estimate of years of life lost for women than men with diabetes could also result from the relatively earlier case detection in Brazilian women [31]. Earlier detection could result in a lead time bias, with less severe disease among many of the affected women. Additionally, if the earlier detection leads to earlier effective treatment of diabetes, this could also lead women to have a lower estimate of years of life lost [32].

Our findings are consistent with diabetes being considered one of the most important epidemic diseases of the 21st century [4]. The challenge of reaching effective diabetes management among Brazilian patients [33] raises a major concern about the burden diabetes will bring. Of importance, this burden will be expressed not only in terms of suffering of those with diabetes and their families, but also in terms of the societal cost of the disease [34,35] and the accompanying economic dampening resulting from the inevitable transfer of societal resources from other uses to support the needed additional health care. Additionally, the increasing cost of treating diabetes, its complications and the other major non-communicable diseases (NCDs) threaten the financial viability of both Brazilian public and private health care systems [34,36].

The extent of the burden we document highlights the importance of engaging society and government in the task of type 2 diabetes prevention and the need to include social determinants and health disparities in actions. Given the current lack of success [37-39] in implementing preventive strategies in Brazil, health care resources spent on diabetes are currently almost exclusively for its treatment. The prevention strategy of frequent diagnostic screening to identify individuals at high risk for type 2 diabetes followed by coaching to improve lifestyle has been shown to be effective [40] and could be implemented to a greater extent in Brazil. We believe our finding of a high lifetime risk can be used in efforts to stimulate individuals to improve lifestyle factors and to periodically monitor glycemic status. Our demonstration of the years of life lost of those with the disease can hopefully be used to stimulate those with diabetes to undertake the actions necessary to prevent complications. However, this high-risk individual strategy [40,41] must be combined with a priority for polices that promote not only better access to and quality of care, especially for those with lower socioeconomic status, but also changes in key dietary risk factors, levels of physical activity, and levels of obesity in the population so as to decrease rates of diabetes incidence [42,43].

Brazil, following the lead of the World Health Organization [44], adopted in 2011 a broad strategy to confront the challenge of rising non-communicable diseases (NCDs) [45] through both prevention and improved management of those currently with disease. Many creative population-based strategies have been initiated in Brazil [46,47]. Worsening risk factor trends, especially those of obesity which extend to the present date [48], despite ongoing implementation of the 2014 Nutritional Guidelines for the Brazilian Population, suggest that much greater effort must be placed in helping Brazilians improve their nutritional habits and increase their level of physical activity [49]. Other Latin American countries have also implemented prevention strategies in recent years [50]. However few studies have evaluated these strategies, including that of diabetes prevention programs targeting of high-risk adults. The increased burden observed on these countries in recent years [51] highlight the importance of such evaluation to establish more effective and sustainable models of prevention.

We hope that this report, along with the many others emphasizing the growing problem of diabetes in Brazil, will stimulate continued discussion of what should be the principal population prevention strategies, how to garner public support for their implementation, and how best to go about their implementation and evaluation.

Potential limitations of our work merit discussion. As previously described, our results are based only on diagnosed diabetes, thus producing conservative estimates for incidence and prevalence, but perhaps overestimates of mortality ratios considering all diabetes, as known cases tend to be more severe. In addition, our incidence analyses are based on age at diagnosis obtained from cross-sectional studies with less than perfect response rates, perhaps introducing some bias. Also, Vigitel is a survey that do not represent the rural areas and smaller cities of Brazil. However, 2013 Vigitel estimates of diabetes prevalence were similar to the nationally representative 2013 National Health Survey (PNS) [52]. Further, the period-based approach to modelling we conducted assumes future diabetes incidence and mortality rates will remain constant over time. Another limitation relates to the representativeness of our estimates of mortality rate ratios. Lacking nationally representative data, we used data from the ELSA-Brasil cohort. Though the ELSA-Brasil sample is not representative of the entire Brazilian population, its use is supported by the fact that its findings in terms of self-reported diabetes are similar to those of Vigitel [23]. In addition, we advise caution when interpreting our years of life lost findings, specifically not considering them as being “due to diabetes”. Although the term years of life lost due to diabetes has become established in the literature, causality cannot be assumed, as in fact we and others merely show the adjusted difference in life expectancy of those with and without diabetes, without a more thorough investigation of causality.

## CONCLUSION

In conclusion, while recognizing these limitations, the adoption of the illness-death model to the Brazilian scenario has allowed us to generate estimates to better understand the diabetes burden in Brazil and, by extension, to add knowledge about middle-income countries. The methodology applied on this study, using data gathered from different sources, can be used to replicate this analysis in other middle-income countries, including those of Latin America. The results from this study, showing novel, easily grasped facets of the diabetes burden expressed at the individual level, will hopefully facilitate health education and advocacy for greater attention to the problems caused by diabetes. The breadth of burden we show demonstrates the extent to which diabetes is a problem for the whole Brazilian population, and thus requires strong, public, population-based prevention policies. In addition, due to the scarcity of similar results from other low and middle-income countries, where more than 80% of the diabetes burden occurs [53], this work contributes to further understanding of the global diabetes burden.

## Additional material

Online Supplementary Document
