# Supplementary material for: Lifetime risk of developing diabetes and years of life lost among those with diabetes in Brazil
Source: J Glob Health. 2021 Jul 3;11:04041. doi: 10.7189/jgh.11.04041 (PMC8284547; doi:10.7189/jgh.11.04041)
Supplement: Online Supplementary Document [file jogh-11-04041-s001.pdf]

Supplemental Material

Appendix S1

Diabetes and Non Diabetes Mortality Rates

The steps to estimate the mortality rate for those with and without diabetes were the following [1,2]:

First, we estimate the overall mortality for those self-reported as white or as black/brown:

For each age (a) and sex (s):

1. We estimate the ethnicity mortality rate ratio ($MRR_{ethnicity}$), comparing those self-reporting as black/brown vs. as white, through Cox regression adjusting only for age and sex.

2. We combine the overall mortality rate for the Brazilian population (M_t), the prevalence of black/brown individuals in this population (P_{bb}) and the $MRR_{ethnicity}$ to estimate the mortality rate of self-reported white (M_w) and black/brown (M_{bb}) individuals.

   Knowing that for each age (a) and sex (s) the mortality rate ratio ($MRR_{ethnicity}$) can be described as:

$$MRR_{\text{ethnicity}}(a,s) = \frac{M_{bb}(a,s)}{M_w\,(a,s)} \;,$$

   we can describe M_t as:

$$M_t(s,a) = P_{bb}\,(s,a) \times M_{bb}(s,a) + \left(1 - P_{bb}(s,a)\right) \times M_w$$

$$M_t(s,a) = P_{bb}\,(s,a) \times M_{bb}(s,a) + M_w(s,a) - M_w(s,a) \times P_{bb}(s,a)$$

$$M_t(s,a) = P_{bb}\,(s,a) \times (M_{bb}(s,a) - \; M_w\,(s,a)) + M_w$$

$$M_t(s,a) = \left[\frac{P_{bb}\ (s,a) \times (M_{bb}(s,a) - M_w\ (s,a)) + M_w\ (s,a)}{M_w\ (s,a)}\right] \times\ M_w\ (s,a)$$

$$M_t(s,a) = \left[P_{bb}(s,a) \times \left(\frac{M_{bb}(s,a)}{M_w\ (s,a)} - 1\right) + 1\right] \times\ M_w\ (s,a)$$

$$M_t(s,a) = M_w \times \left[P_{bb}\ (s,a)\left(MRR_{\text{ethnicity}}\ (s,a) - 1\right) + 1\right]$$

Finally, we estimate the mortality rate for those self-reported as white (M_w):

$$M_w(s,a) = \frac{M_t(s,a)}{\left[P_{bb}(s,a) \times (MRR_{\text{ethnicity}}(s,a) - 1) + 1\right]}$$

and the mortality rate for those self-reported as black/brown (M_{bb}):

$$M_{bb}(s,a) = MRR_{\text{ethnicity}}(s,a) \times M_w(s,a)$$

Then, using a similar approach we estimate in each self-reported ethnicity group the mortality of those with and without diabetes:

3. We estimate the mortality rate ratio (MRR), comparing those with vs. without diabetes, applying Cox regression to ELSA-Brasil cohort data adjusting for body mass index (BMI), waist circumference, schooling, hypertension, income and smoking status, and including interactions terms for age, diabetes, sex and ethnicity.

For each ethnicity, sex and age:

4. We combine the overall mortality rates previously calculated (either M_{bb} or M_{w}) with the corresponding diabetes prevalence (P) and MRR, to estimate the mortality rate of those with (M_1) and without (M_0) diabetes using similar formulas as described before.

Lifetime risk and Years of Life Lost

Cumulative lifetime risk of diabetes, years of life lost and the expected years lived with diabetes were calculated with the Illness-death model [3,4]:

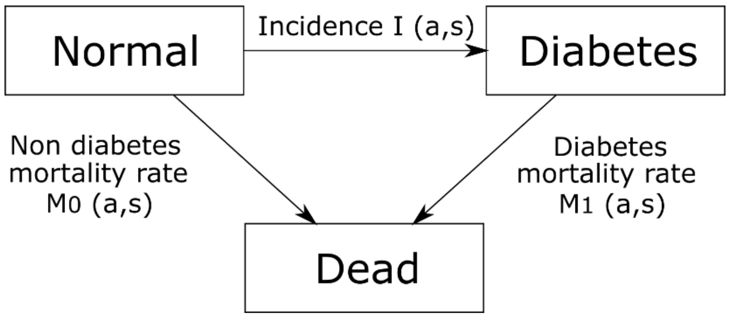

Whiting each ethnicity group, for each sex *s* and age *a*:

Lifetime risk of developing diabetes, before age *a,* and conditional to surviving until *a,* is obtained through the following expression:

$$P_{35}(\mathrm{a}) = \int_{35}^{a} I(t) \times \exp\left(-\int_{35}^{t} I(u) + M_0\,(u)du\right)dt$$

Years of life lost is obtained comparing the life expectancy between individuals of same age *a*, that either develop diabetes (S_1) or remain disease free (S_0).

$$YLL = \int_{a}^{80} (S_0(t) - S_1\,(t))dt$$

The expected years lived with diabetes (S_1), from a certain age *a*, is estimated based on the diabetes mortality rate (M_1):

$$S_1(a) = \exp\left(-\int_a^{80} M_1(u)du\right)$$

The survival of individuals without diabetes (S_0), from a certain age *a*, is based not only on the non-diabetes mortality rate (M_0), but also on the probability of acquiring diabetes in the remaining life time, at a certain age *x,* and therefore decrease the life expectancy from that moment on:

$$S_0(a) = \exp\left(-\int_a^{80} M_0(u) + I(u)\, du\right)$$

$$+ \int_a^{80} I(x) \times exp\left(-\int_a^{x} M_0(u) + I(u)du\right) \times exp\left(-\int_x^{80} M_1(u)du\right) dx$$

It is important to note that our analyses, being derived from cross-sectional survey data on prevalence and incidence, and mortality information centered in the period of these surveys, and being based on the assumption of constant rates into the future, are calculated from a period, and not a cohort, perspective. Therefore, the years of life lost reported in this work is appropriately interpreted as the period expected years of life lost [5].

Uncertainty

We estimated uncertainty by randomly sampling from the distributions of the age-, sex- and ethnicity- specific input parameters incidence, prevalence, and mortality rate ratio. More specifically, we randomly generated a database of 1000 values of diabetes incidence, prevalence, and mortality rate ratio for each ethnicity, sex and age between 35 and 80 assuming independence of all variables. For this purpose, the linear predictors from the logistic regressions used to estimate prevalence or incidence, as well as the log mortality rate ratio, which we estimated as the log hazard ratio through Cox regression, were assumed to follow a normal distribution. The national prevalence of ethnicity groups and all-cause mortality rates are national statistics, and thus were used as constant values. Utilizing each of the 1000 databases

we performed all the calculations described to estimate lifetime risk of developing diabetes, years of life lost among those with diabetes, and years lived with diabetes, and from these results we identified the 2.5th and 97.5th percentiles as the confidence intervals.

All the statistical codes used to generate our estimates can be provided upon request to the corresponding author (paula.abracco@gmail.com).

Table S1 – Socioeconomic and clinical characteristics of the 2017-2019 VIGITEL samples analyzed

	Women			Men		
	White	Black/Brown		White	Black/Brown	
**Characteristics**	**%**	**%**	**p-value***	**%**	**%**	**p-value***
Self-assessed health condition			< 0.001			< 0.001
Good or Very Good	69.5	59.5		75.4	68.6	
Regular	25.9	34.6		21.7	27.9	
Poor or Very Poor	4.6	5.9		2.8	3.5	
Education			< 0.001			< 0.001
Incomplete High School	26.3	28.4		24.9	30.0	
Completed High School	31.5	42.9		33.4	45.0	
More than High School	42.3	28.7		41.7	25.0	
Private health insurance	57.6	40.2	< 0.001	54.1	38.4	< 0.001
Governmental cash transfer ("Bolsa Família")	4.6	9.8	< 0.001	3.3	7.6	< 0.001
Intake of five or more ultraprocessed food group	14.8	15.9	0.248	20.0	23.8	0.007
Regular intake of fruits or vegetables (5 or more times per week)	45.1	35.1	< 0.001	32.8	24.3	< 0.001
Smoking	7.4	7.1	0.346	12.8	11.4	0.039
Obesity	17.8	21.2	< 0.001	19.0	19.3	0.695
Hypertension	25.8	25.4	0.505	21.1	20.9	0.756
Last glucose test			0.051			0.002
Within 6 months	61.5	59.7		56.2	52.0	
6 months to 1 year	20.2	18.7		17.6	17.4	
1 to 2 years	8.3	9.8		10.7	10.3	
2 to 3 years	2.1	2.7		4	3.5	
More then 3 years	3.2	3.0		3.8	5.2	
Never	1.2	1.4		2	3.6	
Don't Remember	3.5	4.6		5.8	7.9	

***chi-square test**

References

[1] Jacobs E, Hoyer A, Brinks R, Kuss O, Rathmann W. Burden of Mortality Attributable to Diagnosed Diabetes: A Nationwide Analysis Based on Claims Data From 65 Million People in Germany. Diabetes Care 2017;40:1703–9.

[2] Bracco PA, Gregg EW, Rolka DB, Schmidt MI, Barreto SM, Lotufo PA, et al. A nationwide analysis of the excess death attributable to diabetes in Brazil. J Glob Health 2020;10.

[3] Brinks R, Hoyer A, Kuss O, Rathmann W. Projected Effect of Increased Active Travel in German Urban Regions on the Risk of Type 2 Diabetes. PLoS One 2015;10:e0122145.

[4] Carstensen B. Epi: Years of Life Lost (YLL) to disease Diabetes in DK as example (2017). R package version 2.19. Available: https://mran.microsoft.com/snapshot/2017-04-22/web/packages/Epi/vignettes/yll.pdf. Accessed: 20 November 2020.

[5] Murray CJ, Mathers CD, Salomon JA, Lopez AD. Health gaps: an overview and critical appraisal. Summary Measures of Population Health-Concepts, Ethics, Measurement and Applications. Geneva: WHO. 2002;233-244.
